# Resistance to PD-1/PD-L1 blockade cancer immunotherapy: mechanisms, predictive factors, and future perspectives

**DOI:** 10.1186/s40364-020-00212-5

**Published:** 2020-08-26

**Authors:** Jin-Yu Sun, Dengke Zhang, Songquan Wu, Min Xu, Xiao Zhou, Xiao-Jie Lu, Jiansong Ji

**Affiliations:** 1 The First College of Clinical Medicine, the First Affiliated Hospital of Nanjing Medical University, Nanjing Medical University, Nanjing, China; 2 Key Laboratory of Imaging Diagnosis and Minimally Invasive Intervention Research, Lishui Hospital of Zhejiang University/ The Fifth Affiliated Hospital of Wenzhou Medical University/ Clinical Medicine of Center Hospital of Lishui College, Lishui, 323000 China; 3 College of Medicine, Lishui College, Lishui, 323000 China; 4 Department of General Surgery, The First Affiliated Hospital of Nanjing Medical University, Nanjing, China; 5 Department of radiology, Affiliated Lishui Hospital of Zhejiang University, Lishui, 323000 China

**Keywords:** PD-1/PD-L1 blockade, Cancer immunotherapy, Resistance

## Abstract

PD-1/PD-L1 blockade therapy is a promising cancer treatment strategy, which has revolutionized the treatment landscape of malignancies. Over the last decade, PD-1/PD-L1 blockade therapy has been trialed in a broad range of malignancies and achieved clinical success. Despite the potentially cure-like survival benefit, only a minority of patients are estimated to experience a positive response to PD-1/PD-L1 blockade therapy, and the primary or acquired resistance might eventually lead to cancer progression in patients with clinical responses. Accordingly, the resistance to PD-1/PD-L1 blockade remains a significant challenge hindering its further application. To overcome the limitation in therapy resistance, substantial effort has been made to improve or develop novel anti-PD-1/PD-L1 based immunotherapy strategies with better clinical response and reduced immune-mediated toxicity. In this review, we provide an overview on the resistance to PD-1/PD-L1 blockade and briefly introduce the mechanisms underlying therapy resistance. Moreover, we summarize potential predictive factors for the resistance to PD-1/PD-L1 blockade. Furthermore, we give an insight into the possible solutions to improve efficacy and clinical response. In the following research, combined efforts of basic researchers and clinicians are required to address the limitation of therapy resistance.

## Background

Immunotherapy is a validated and significant cancer treatment strategy, which eliminates tumors by normalizing the anti-tumor immune responses [1, 2]. Over the last decade, cancer immunotherapy has revolutionized the treatment landscape of malignancies and achieved clinical success, especially in immune checkpoint inhibitors [3].

Programmed death-1 (PD-1) is a class of receptor expressed on the T cell surface, which could down-regulate the immune system by abrogating T cell receptor-induced signals and preventing antigen-mediated T cell activation [4]. The interaction between PD-1 and its ligand (programmed death-ligand 1, PD-L1) plays an essential role in maintaining self-tolerance and avoiding autoimmune diseases. However, PD-1/PD-L1 could also prevent the activation of T cells in the tumor and thus result in immune resistance [5].

PD-1/PD-L1 blockade is a breakthrough in cancer immunotherapy, and it has been trialed in a broad range of malignancies in the preclinical or clinical stage, including melanoma [6], Hodgkin’s lymphoma [7], breast cancer [8, 9], non-small cell lung cancer (NSCLC) [10], as well as hepatocellular carcinoma [11, 12]. Despite the long-term, potentially cure-like clinical benefits, therapy resistance remains a significant challenge for the further application of PD-1/PD-L1 blockade therapy. Only a minority of patients (20–30%) in general are estimated to experience a positive response to PD-1/PD-L1 blockade therapy [13–15], and the primary or acquired resistance might eventually lead to cancer progression in patients with clinical response [16, 17].

In this review, we provide an overview on the resistance to PD-1/PD-L1 blockade and its underlying mechanisms. Moreover, we summarize potential predictive factors for the resistance to PD-1/PD-L1 blockade. Furthermore, we give an insight into the possible solutions to improve efficacy and clinical response of PD-1/PD-L1 blockade therapy.

## Resistance to PD-1/PD-L1 blockade therapy

Checkpoint inhibitors targeting PD-1 or PD-L1 could disturb the interaction between PD-1 and PD-L1, which would preserve anti-tumor properties of T cells, withdraw immune escape, and normalize their ability to induce tumor cell death. Currently, PD-1/PD-L1 blockade has shown sustained survival benefits in multiple malignancies and is at the forefront of cancer immunotherapy [18]. However, just as tumor cells can avoid immune evasion, several cancers may evolve to resist PD-1/PD-L1 blockade therapy. Clinical evidence indicated that even for patients with tumors highly positive for PD-L1, more than 50% of them might not respond to PD-1/PD-L1 blockade [19]. Due to tumor heterogeneity and many other reasons, clinical responses vary largely across different tumor entities. The objective response rate was 30–45% in melanoma [20], 15–20% in NSCLC [21], 13% in head and neck carcinoma [22], and 22–25% in kidney cancer [23]. Besides, for most patients experiencing initial clinical response, acquired resistance remains another problem, which would lead to cancer progression or relapse after a few years [16, 24].

Many studies have demonstrated that anti-PD-1 therapy can significantly improve survival outcomes for patients with metastatic or unresectable melanoma [25, 26]. However, only a small number of patients (approximately 8–15%) could achieve a complete response. In a recent phase I trial of atezolizumab (anti-PD-L1) involving 45 patients with metastatic melanoma [27], the overall response rate was 30% among 43 efficacy evaluable patients, and the median response duration was 62 months. Moreover, in another study on the long-term outcomes of melanoma patients receiving anti-PD-1 therapy, complete responses were only observed in 102 of 396 patients (25.8%) [6]. After a median follow-up of 21.1 months, 72.1% of patients were alive without additional melanoma therapy. Additionally, in the retreated patients after disease progression, the response was only observed in 14.7% retreated patients receiving single-agent PD-1 blockade therapy, and 25.0% of patients escalated to PD-1 blockade plus ipilimumab therapy. In this cohort, most complete responses were durable with the treatment failure rate of 27% at three years, while the response to retreatment remained relatively infrequent with a response rate of 14.7% for patients with single-agent PD-1 blockade therapy [6]. Moreover, in a phase II study of pembrolizumab on 39 patients with advanced adrenocortical carcinoma, the objective response was observed in 23% of patients with a disease control rate of 52% after a median follow-up of 17.8 months [28].

Interestingly, the response rate of some malignancies is relatively high in hematological malignancies. For example, for patients with relapsed or refractory classical Hodgkin lymphoma, tislelizumab (anti-PD-1) achieved an objective response rate of 87.1% and a complete response of 62.9%, in a phase II, single-arm, multicenter study [7]. Similarly, the complete response rate of camrelizumab (anti-PD-1) was 28.0%, with a partial remission rate of 48.0% [29].

## Mechanisms underlying the resistance to PD-1/PD-L1 blockade

Since therapy resistance remains a significant limitation of PD-1/PD-L1 blockade in clinical practice, interest is growing in understanding the mechanisms underlying the resistance. The response to PD-1/PD-L1 blockade relies on a pre-existing immune response and determinants of adaptive immunity. Currently, multiple factors have been discovered to be involved in the efficacy of PD-1/PD-L1 blockade therapy, such as tumor immunogenicity, T cell function, PD-L1 expression, tumor microenvironment, and so forth.

### The lack of tumor antigens

The genetic alterations are central in the oncogenic process, which could lead to tumor immunogenicity and provide an opportunity for cancer immunotherapy [17]. Tumor immunogenicity is positively associated with the ability of the T cell to recognize tumor cells, which is essential for the anti-tumor effect of PD-1/PD-L1 blockade. However, the lack of tumor antigen will significantly impede the recognition ability of T cells and eventually result in the failure of immunotherapy.

Microsatellites are prone to DNA replication errors, which will usually be repaired in normal cells [30]. However, in tumors with mismatch repair (MMR) deficiency, these errors will accumulate, which eventually result in a large number of mutations and lead to microsatellite instability (MSI) [30]. Importantly, high MSI positively contributes to increased neoantigen production, greater immunogenicity, and a more robust immune response [31]. Moreover, the resultant high tumor mutation burden would contribute to tumor immunogenic and enhance the response to PD-1/PD-L1 blockade therapy [32, 33].

Multiple studies have demonstrated that the tumor mutation burden is positively correlated with neoantigen burden as well as response to immunotherapy [34, 35]. For example, in colorectal cancer with MMR deficiency, which usually exhibits a high tumor mutation burden, anti-PD-1 therapy showed a higher response rate and better survival outcome compared to other subtypes with MMR proficiency [36–38]. Yarchoan et al. [38] analyzed the objective response rates of PD-1/PD-L1 blockade therapy for the corresponding tumor mutation burden in various cancers, and their results showed that the mutation burden was closely associated with the objective response rate [38].

Moreover, pancreatic cancer generally exhibits a lower mutation load compared with other solid tumors, and therefore, PD-1/PD-L1 blockade is usually ineffective for those patients and fails to improve their survival outcomes. Nevertheless, in pancreatic cancer patients harboring an MMR deficiency, they appear to be responsive to PD-1/PD-L1 blockade therapy. MMR deficiency significantly increases the somatic mutation rate, which could be translated into neoantigens and recognized by the immune system, thus making these patients responsive to PD-1/PD-L1 blockade therapy [36, 39]. Accordingly, pembrolizumab has been approved for selected cancer patients with MMR deficiency.

### T cell dysfunction

Effective PD-1/PD-L1 blockade therapy relies on the T cell function, and any disruption in the processes of T cell immune function will result in the failure of PD-1/PD-L1 blockade therapy. A recent review article by Ren et al. has provided an in-depth insight into the mechanisms underlying the T cell dysfunction-mediated resistance, with a focus on T cell recognition, activation, differentiation, infiltration, depletion, as well as chemotaxis [40].

Antigen presentation is a critical process for the tumor antigens identification by initial T cells. Beta-2-microglobulin (B2M) is a significant HLA1 molecule whose mutation will hinder tumor antigen presentation and result in therapy resistance [41–43]. Zaretsky et al. [43] analyzed biopsy samples from patients with metastatic melanoma receiving pembrolizumab who exhibited disease progression after an initial tumor regression, and they found a truncating mutation in the *B2M* gene. In the following research, Gettinger et al. [41] identified acquired homozygous loss or downregulation of B2M in lung patients with resistance to PD-1/PD-L1 blockade. To further explore the role of B2M in mediating resistance, they knocked out the *B2M* gene in immunocompetent lung cancer mice by CRISPR technology, and the loss of *B2M* resulted in the resistance to PD-1/PD-L1 blockade [41]. Additionally, *B2M* mutation-induced resistance primarily occurred in an environment of activated PD-1 positive T cell infiltration, which suggested that resistance to PD-1/PD-L1 blockade therapy might be particularly common in patients with high PD-1 positive T cell [44].

Moreover, T cell activation is another critical process for PD-1/PD-L1 blockade therapy. After blocking PD-1/PD-L1, tumor cells can still counteract the activity of immune checkpoints and activate additional inhibitory pathways via expression of other immune checkpoints and their ligands within the tumor immune microenvironment [45]. For example, T cell immunoglobulin mucin-3 (Tim-3) is another type of immune checkpoint receptor expressed on tumor-infiltrating lymphocytes. In human head and neck squamous cell carcinoma tumor-infiltrating lymphocytes, PD-1 blockade was demonstrated to up-regulate Tim-3 expression, which inhibited T cells activation and contributed to Tim-3-mediated escape from PD-1 blockade in the tumor microenvironment via PI3K/Akt pathway [46].

### PD-1 or PD-L1

Physiologically, interactions between PD-1 and PD-L1 block T cell activation pathways related to the immune response against specific antigens, and the expression of PD-1 or PD-L1 has gained importance as a significant player in regulating the response to PD-1/PD-L1 blockade therapy. PD-1 and PD-L1 are up-regulated in the tumor immune microenvironment of various malignancies, which is considered as a strategy to evade immunosurveillance and imposes a significant barrier of the anti-tumor immune response [47]. Importantly, PD-L1 primarily exhibits two distinct expression patterns: on tumor cells or on tumor-infiltrating immune cells. PD-L1 expression on immune cells reflects the adaptive regulation meditated by IFN-γ, which is accompanied by increased effector T cells as well as tumor-infiltrating lymphocytes effector T cells. Differently, the expression of PD-L1 on tumor cells is less prevalent, and it indicates the epigenetically dysregulated PD-L1 gene, which is correlated with reduced immune infiltration, sclerotic or desmoplastic stroma, as well as mesenchymal molecular features [48].

Multiple studies have revealed a significantly higher objective response rate in tumor PD-L1 positive patients than PD-L1 negative subgroups, together with an improved progression-free and overall survival [21, 49–51]. Kowanetz et al. [48] observed that atezolizumab (anti-PD-L1) achieved an objective response rate of 40% in patients with high PD-L1 levels on tumor cells alone and of 22% in those with a high expression on immune cells alone. Although these observations indicated that the functional importance of PD-L1 expression in regulating PD-1/PD-L1 blockade-induced T cell response, the mechanistic significance of PD-L1 on tumor cells or immune cells remains vague.

### Noncoding RNAs

A large amount of microRNAs (miRNAs) and some long noncoding RNAs (lncRNAs) have emerged as players in regulating tumor immunity [52–54] and resistance to PD-1/PD-L1 blockade therapy [55].

Recently, Huber et al. [56] identified a panel of circulating miRNAs (miR-146a, miR-155, miR-125b, miR-100, let-7e, miR-125a, miR-146b, miR-99b), which were associated with phenotypic and functional features of myeloid-derived suppressor cells (MDSCs) in melanoma patients. Importantly, MDSCs are a subclass of immature myeloid cells pathologically associated with cancer and play an inhibitory role against anti-tumor T cell immunity [57]. The transcriptional analysis showed that these miRNAs could facilitate the conversion of monocytes into MDSCs by melanoma extracellular vesicles, and the expression level of these miRNA was up-regulated in circulating CD14^+^ monocytes and tumor samples, which was associated with myeloid cell infiltration and could predict the resistance to PD-1 blockade therapy [56].

Moreover, Hu et al. [58] revealed the role of oncogenic lncRNA for kinase activation (LINK-A) in losing antigenicity and evading immune checkpoints and demonstrated lncRNA-dependent antigenicity downregulation and intrinsic tumor suppression. For patients with triple-negative breast cancer and resistant to PD-1 blockade therapy, they showed up-regulated LINK-A levels and downregulated peptide-loading complex components. The analysis suggested that LINK-A expression could attenuate protein kinase A-mediated phosphorylation of the E3 ubiquitin-protein ligase TRIM71 via facilitating the crosstalk between phosphatidylinositol- [3–5]-trisphosphate and inhibitory G-protein-coupled receptor pathways. Consequently, LINK-A could contribute to the degradation of the antigen peptide-loading complex and up-regulate intrinsic tumor suppressors [58].

### Gut microbiome

The gut microbiome is a complex system composed of more than 100 trillion microorganisms, which has been demonstrated to regulate the efficacy and toxicity of cancer immunotherapy. Many studies have reported the influence of the gut microbiome on cancer immunotherapy, and the therapeutic response of PD-1/PD-L1 blockade therapy can be improved or diminished via gut microbiome modulation.

In mice models with distinct microbiome, a significantly different response to PD-1/PD-L1 blockade therapy was observed. For example, melanoma mice with an increased *Bifidobacterium* species in the gut microbiome exhibited an effective response to PD-1 blockade therapy [59]. Similarly, antibiotic administration was reported to reduce the diversity and aggravate dysbiosis of the gut microbiome, thus influencing the clinical response to PD-1/PD-L1 blockade in tumor-bearing mice as well as cancer patients [60–62].. Compared to patients without antibiotic treatment, the oral antibiotic application could significantly diminish the clinical benefit of PD-1/PD-L1 blockade therapy and decrease progression-free survival and overall survival [61].

Therefore, dysbiosis of the gut microbiome is considered as one of the putative mechanisms underlying poor response to PD-1/PD-L1 blockade therapy, and the dual-directional modulation of the gut microbiome on cancer immunotherapy is increasingly revealed. However, it is still unclear how gut microbiome regulates therapy response, and whether a specific bacterial taxa or gut microbiome as a whole plays a primary role remains largely unclear. Further research is required to provide a more in-depth understanding of the underlying mechanisms.

## Predictive factors for PD-1/PD-L1 blockade therapy

Despite the clinical success achieved in PD-1/PD-L1 blockade across multiple cancers, the knowledge concerning therapy selection criteria is relatively limited. Considering the potential adverse events and high cost of immune checkpoint inhibitor agents, there is a substantial need to identify predictive factors to select patients likely to benefit from this therapy. Currently, apart from the functional status of immune cells [63–66] or tumor infiltrating lymphocytes [67], multiple factors have been identified to predict the response to PD-1/PD-L1 blockade therapy, such as PD-1/PD-L1 expression, antigen recognition, gut microbiome, and so forth (Table 1).

**Table 1:** Predictive factors for PD-1/PD-L1 blockade therapy

Tumor type	Agent	Predictive factor	Reference
Non-small cell lung cancer	Atezolizumab	PD-L1	[68]
Multiple cancers	Pembrolizumab	PD-L1	[69]
Colorectal cancer	Nivolumab	MMR / MSI	[70]
Urothelial carcinoma	Atezolizumab	TMB	[71]
Urothelial carcinoma	Atezolizumab	TMB	[72]
Urothelial cancer	Atezolizumab	TMB	[73]
Melanoma	Anti-PD-1 therapy	Gut microbiome	[74]
Melanoma	Anti-PD-1 therapy	Gut microbiome	[75]
Melanoma	Anti-PD-1 therapy	Gut microbiome	[61]

MMR: mismatch repair; MSI: microsatellite instability; TMB: tumor mutation burden

### PD-1 or PD-L1 expression

Inhibiting the PD-1 pathway-mediated immune suppression is the basis and premise of PD-1/PD-L1 blockade therapy. Accumulating research has suggested that PD-L1 is a biomarker to predict therapeutic response to PD-1/PD-L1 blockade across multiple tumor types [15]. For example, atezolizumab achieved overall survival benefit across all PD-L1 expression subgroups in NSCLC patients, while those with high PD-L1 expression experienced a more substantial survival benefit [76]. Currently, PD-L1 testing is recommended as a predictive test for NSCLC [68], urothelial carcinoma [77], or head and neck cancers [78], and so forth.

Ott et al. [79] assessed the predictive value of PD-L1 expression in patients with advanced solid tumors receiving pembrolizumab, and the analysis showed that tumors with higher PD-L1 expression and tumor mutation burden were significantly associated with higher response rate and more prolonged progression-free survival. Heat map analysis revealed a close correlation between PD-L1 expression and a broader pattern of coregulated gene expression, which involved cytokine recruitment of T cells, T cell activation markers, as well as antigen presentation. Also, the regression meta-analysis demonstrated that PD-L1 expression level was positively associated with objective response rate (*P* = 0.018) as well as progression-free survival (*P* = 0.005, 72).

Moreover, NCT02853305 and NCT02807636 evaluated the efficacy of pembrolizumab or atezolizumab as first-line treatment, and the current data showed reduced survival in patients with low expression of PD-L1. Accordingly, it is advised that pembrolizumab or atezolizumab should be used for adult patients with a relatively high PD-L1 expression (PD-L1 expression of ≥5% for atezolizumab, and a combined positive score of ≥10 for pembrolizumab). However, the efficacy of PD-1/PD-L1 blockade therapy as first-line therapy for advanced urothelial carcinoma still remains unclear [15, 69].

Importantly, PD-L1 positive only is not a predictive factor for the response to PD-1/PD-L1 blockade, since multiple factors are involved in the PD-1/PD-L1 blockade therapy. In a study on 46 patients with metastatic melanoma receiving pembrolizumab, pre-existing CD8^+^ T cells were demonstrated as a prerequisite for the tumor regression after PD-1/PD-L1 blockade therapy [80]. Besides, in advanced adrenocortical carcinoma, tumor PD-L1 expression status was not associated with therapy response [28]. Additionally, it was reported that PD-L1 expression on tumor cells was not associated with therapy response in resected head and neck squamous cell cancer [81]. Additional investigation is required to illustrate the mechanisms accounting for the difference.

### Antigen recognition

Antigen recognition plays a vital role in initiating the adaptive immune response, while the lack of tumor antigens significantly impedes the response to PD-1/PD-L1 blockade therapy.

Currently, the FDA has approved pembrolizumab to treat unresectable solid tumors with high MSI or MMR deficiency [82]. In a study on recurrent or metastatic colorectal cancer patients with MMR deficiency or high MSI, nivolumab showed an objective response rate of 31.1, and 69% of the patients had a disease control rate of ≥12 weeks, which indicated that patients with high MMR deficiency or high MSI might exhibit better responses to PD-1/PD-L1 blockade therapy [40, 83]. Interestingly, the responses of tumors with MMR-deficient are highly variable, and approximately half are resistant to PD-1/PD-L1 blockade therapy. Mandal et al. [32] revealed that MSI and the resultant mutation load were responsible for the variable response to PD-1 blockade therapy in MMR-deficiency tumors, and the response degree was significantly correlated with the degree of insertion-deletion mutation load.

Several studies have revealed the association between tumor mutation burden and the response to PD-1/PD-L1 blockade therapy [70, 71]. Mariathasan et al. [72] examined samples from patients with metastatic urothelial cancer receiving atezolizumab treatment and identified high neoantigen and tumor mutation burden as major determinants of clinical outcome. Their results showed that the tumor mutation burden was closely correlated with the response in the excluded and inflamed phenotypes.

### Gut microbiome composition

Clinical experiments on the human gut microbiome have identified several specific bacteria genres that play important roles in human immunity and can be used as prognostic biomarkers for clinical response to PD-1/PD-L1 blockade therapy [73].

Based on the gut microbiome analysis of melanoma patients receiving PD-1 blockade, Gopalakrishnan et al. [84] found that patients with prolonged progression-free survival showed a higher multiplicity of bacteria, and *Clostridiales*, *Ruminococcaceae*, and *Faecalibacterium* were abundant in therapy responders. Moreover, Matson et al. [74] evaluated the baseline stool samples from patients with metastatic melanoma before PD-1/PD-L1 blockade treatment, and the results showed that commensal microbial composition was significantly associated with the clinical response: *Bifidobacterium longum*, *Collinsella aerofaciens*, and *Enterococcus faecium* were more abundant in responders. Similarly, in patients with epithelial tumors, Routy et al. [60] revealed that *Akkermansiacea muciniphila* and *Enterococcus hirae* were significantly abundant in those with better clinical response (progression-free survival > 3 months). All these results indicate that gut microbiome composition may be a potential determinant of therapy response and might be used as a predictive factor. In the following research, more studies are needed to validate the predictive value of gut microbiome in larger cohorts and explore their efficiency in the context of various types of tumors.

## Future perspectives

**Fig. 1:**
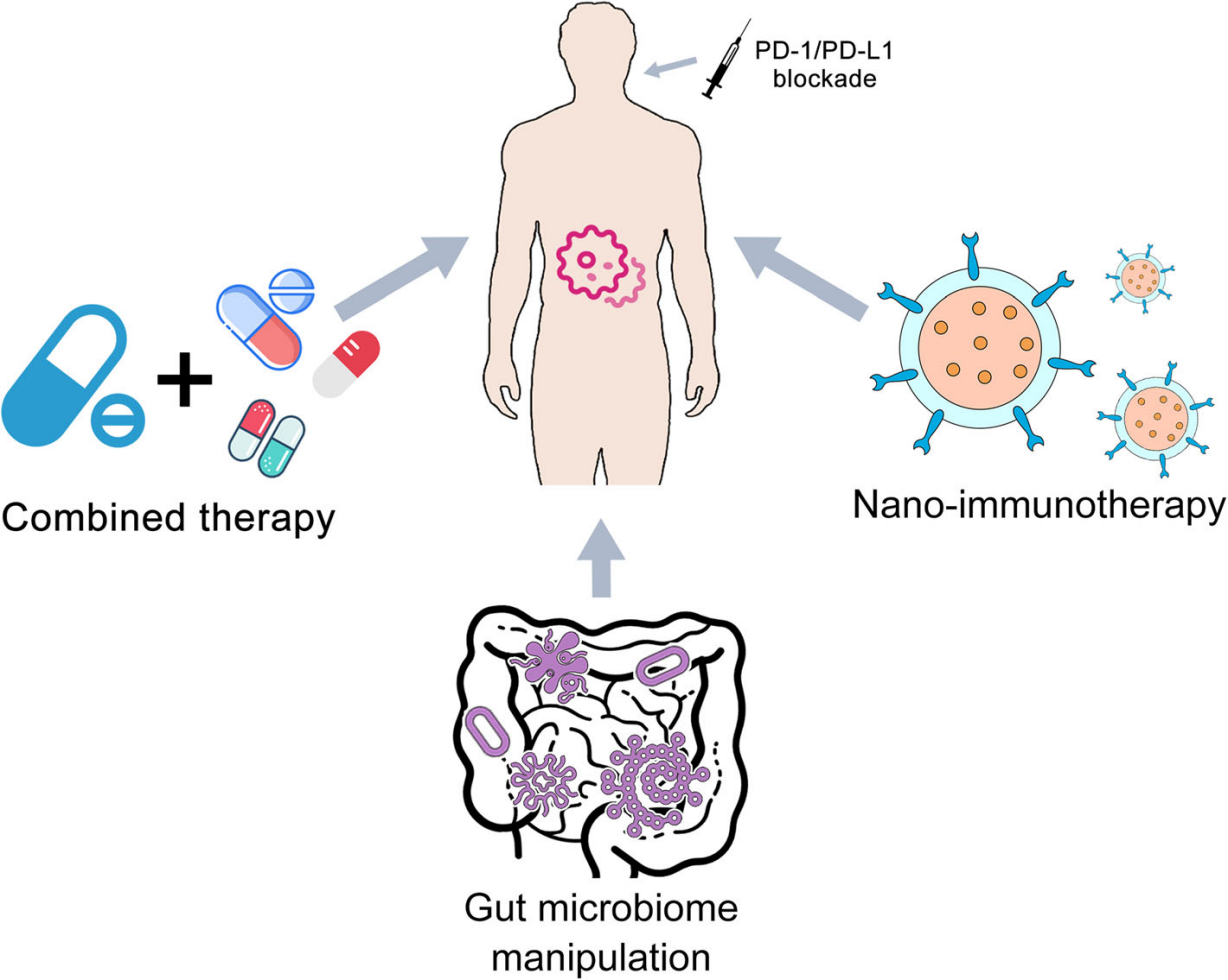
**Overview on the strategies to improve the resistance to PD-1/PD-L1 blockade therapy.** Multiple strategies have been proposed to improve the resistance to PD-1/PD-L1 blockade therapy, including combined therapy, nano-immunotherapy, gut microbiome manipulation, and so forth

Immunotherapy is one of the most promising cancer treatment strategies, and it has revolutionized the landscape of cancer management over the last decade. However, together with the costly and time-consuming trial-and-error approach, the limited therapy response remains a tricky problem, which hinders the further application of PD-1/PD-L1 blockade. To overcome therapy resistance and potential adverse events, substantial effort has been made on developing novel anti-PD-1/PD-L1 based immunotherapy strategies with better clinical response and limited immune-mediated toxicity (Figs. 1, 84).

Since the interaction between cancer and the immune system is complex and involves multiple factors, strategies in combination with multiple agents are likely to achieve better clinical outcomes compared with single-agent administration. A large number of studies have revealed that combined therapy is an effective therapeutic strategy against cancers. For example, transforming growth factor β (TGFβ)-blocking agents concomitantly with combined PD-1/PD-L1 blockade combined provides a clinically feasible strategy to improve efficacy and reduce toxicity. Mariathasan et al. [72] revealed that metastatic urothelial cancer with up-regulated TGFβ signaling before treatment responded poorly to PD-1/PD-L1 blockade therapy. The tumors with dense collagen fibrils could trap T cells in the stromal compartment, thus preventing them from playing their functions. In preclinical experiments on mice with immune-excluded phenotype, they demonstrated that the co-administration of PD-L1 blockade and TGFβ-blocking agents could reduce TGFβ signaling, facilitate T cell infiltration, and achieve active anti-tumor immunity and tumor regression. Similarly, the combination of PD-1/PD-L1 blockade with tumor necrosis factor inhibitor [85, 86], metformin [87], anti-VEGF agents [88], or other immune checkpoint inhibitors (e.g., CXCR4 [89]) has been demonstrated as a clinically feasible strategy with improved anti-tumor efficacy and reduced toxicity.

PD-1/PD-L1 blockade usually acts on the whole host immune system instead of site­specifically targeting tumor­specific immune cells, while nanomedicine technology provides a powerful tool to selectively deliver immune checkpoint inhibitor agents to tumors or lymphoid organs, using drug-loaded nanoparticles (usually 5 to 1,00 nm in diameter) [90]. Recent studies suggest that the PD-1/PD-L1 antibody could be conjugated or modified on the surface of nanoparticles, which could maintain their stability, enhance efficiency, and minimize the toxicity of PD-1/PD-L1 blockade [7, 91, 92]. For example, in gastric cancer cells, the PD-L1 blockade-conjugated nanoparticles contributed to significantly higher cellular uptake and achieved more effective inhibition of PD-L1 expression compared with the control groups [93]. Moreover, in patients with metastatic triple-negative breast cancer, the co-administration of nab-paclitaxel plus PD-L1 blockade (atezolizumab) prolonged progression-free survival [94]. Owing to the success in previous research [95], clinical trials on nano-immunotherapy are currently underway, such as NCT03589339, and NCT03684785. These clinical trials should provide substantial evidence for the combination of nanomedicine and PD-1/PD-L1 blockade in the next few years.

Furthermore, accumulating evidence has demonstrated that gut microbiome significantly impacts the efficacy of cancer immunotherapy, which in turn indicates that the manipulation of the gut microbiome could latently affect the response to PD-1/PD-L1 blockade therapy [96–98]. Currently, antibiotic application, fecal microbiota transplantation (FMT), and diet regulation are considered as practical approaches to manipulate gut microbiome. For example, FMT from patients with a positive response to germ-free or antibiotic-treated mice could improve tumor control, augment T cell responses, and ameliorate the anti-tumor effects of PD-1 blockade. In contrast, the transplantation from resistant patients did not result in improvement [60]. Similarly, responses to PD-L1 blockade are distinct in mice with different commensal microbes, and the positive response of mice with advantageous gut microbiome can be transplanted to mice with negative responses by FMT or co-housing [59].

## Conclusions

Despite the success across multiple types of cancers, only a minority of patients are estimated to exhibit a positive response to PD-1/PD-L1 blockade therapy, and the primary/acquired resistance might eventually lead to progression in patients with clinical responses. The limitation in clinical response impairs the efficacy and hinders its further application. Since the understanding of the mechanisms underlying therapy resistance remains vague, only a few therapeutic options are available for those patients. Currently, illustrating the determinants of response or resistance is significant to accelerate improving survival outcomes and developing improved treatment options for cancer patients. To better realize the therapeutic potential of PD-1/PD-L1 blockade therapy, it is essential to identify predictive biomarkers for therapy response, develop novel therapeutic strategies, and improve therapeutic strategies in combination with other agents. In the following research, combined efforts of basic researchers and clinicians are required to address the PD-1/PD-L1 blockade therapy resistance.

## Data Availability

Not applicable.

## Abbreviations

PD-1: programmed death-1
PD-L1: programmed death-ligand 1
NSCLC: non-small cell lung cancer
MMR: mismatch repair
MSI: microsatellite instability
B2M: beta-2-microglobulin
Tim-3: T cell immunoglobulin mucin-3
miRNAs: microRNAs
lncRNAs: long noncoding RNAs
MDSCs: myeloid-derived suppressor cells
TGFβ: transforming growth factor β
FMT: fecal microbiota transplantation
